# Delayed adverse reaction to a natural dermal filler mimicking salivary gland neoplasia

**DOI:** 10.1186/s42269-022-00791-3

**Published:** 2022-04-11

**Authors:** Nasreen Alli, Marshall Murdoch, Shabnum Meer

**Affiliations:** 1 KwaMashu Community Health Centre, Durban, KwaZulu Natal South Africa; 2 Knysna, South Africa; 3 Department of Oral Pathology, Faculty of Health Sciences, University of the Witwatersrand, WITS, Private Bag 3, Johannesburg, 2050 South Africa

**Keywords:** Dermal fillers, Soft tissue injectables, Hyaluronic acid, Adverse reaction natural dermal fillers, Adverse reaction mRNA COVID-19 vaccines

## Abstract

**Background:**

Cosmetic dermal fillers are a sought-after procedure globally. However, despite the safety claims of filler materials by the manufacturers, adverse reactions still occur.

**Case presentation:**

This case report is of a 66-year-old female who presented with a late-onset complication of a hyaluronic acid dermal filler that clinically mimicked a salivary gland neoplasm. The patient presented with firm peri-oral swellings of short duration that clinically mimicked a pleomorphic adenoma and mucoepidermoid carcinoma. The diagnosis was that of a foreign-body granulomatous response to dermal fillers. Although other mimics of a similar nature are reported a knowledgeable clinician, careful choice of filler material, knowledge of the product, thorough pre-procedural history taking and post-procedural patient follow-up can drastically minimize a possible misdiagnosis. The reaction was treated with a combination of hyaluronidase, betamethasone and 5-flurouracil intra-lesional injections monthly for 11 consecutive months, with total clinical resolution.

**Conclusions:**

Patient education of the procedure, product name and the possibility of an adverse reaction occurring, even years later or at a site distant to the initial site of placement, is vital. Further, we review the recent reported adverse association of the new mRNA COVID-19 vaccines and dermal filler placement.

## Background

The twenty-first-century global population is remarkably inclined toward aesthetic procedures that target the alteration, enhancement and maintenance of facial appearance. Statistics from the American Society of Plastic Surgeons (ASPS) show that soft tissue filler rose rapidly in popularity and has been the second most performed minimally invasive cosmetic procedure every single year since 2007 (http://www.plasticsurgery.org). Both the increasing demand for facial aesthetic procedures and the growing market for dermal filler products have resulted in a parallel spike in complications (Dyke et al. 2010). Despite the safety and tissue compatibility claims, adverse reactions continue to be reported (Alijotas-Reig et al. 2013a). This unusual case explores together with a literature review a late-onset complication of a dermal filler that clinically mimicked a salivary gland neoplasm.

## Case presentation

### Clinical findings

A 66-year-old female patient presented with firm, slightly warm peri-oral swellings of 2 months duration. Examination revealed palpable, tender, indurated and fixed subcutaneous masses in the upper lip, both the nasolabial folds and especially the buccal aspect of the angle of the mandible. The overlying skin appeared normal, yet intra-orally the buccal mucosa was erythematous. Ultrasound-guided fine needle aspiration (FNA) biopsy from the upper left lip showed abundant extracellular pale pink staining mucinous-type matrix material with interspersed foamy cells, reported as being suggestive of a mucoepidermoid carcinoma or pleomorphic adenoma. She was a non-smoker, with no allergies nor significant family history. There was no history of trauma, difficulty with filler placement, prior dental procedures, local or generalized infection, including COVID-19 infection prior to the development of the inflammatory response nor vaccination. In the absence of a history of dermal filler placement, the presence of multiple indurated nodules, which appeared clinically infiltrative, the rapid development of the lesions, the FNA findings and the frequent and similar occurrence of salivary gland neoplasia at this site the clinical differential diagnosis was that of a salivary gland neoplasm.

### Pathological findings

**Fig. 1:**
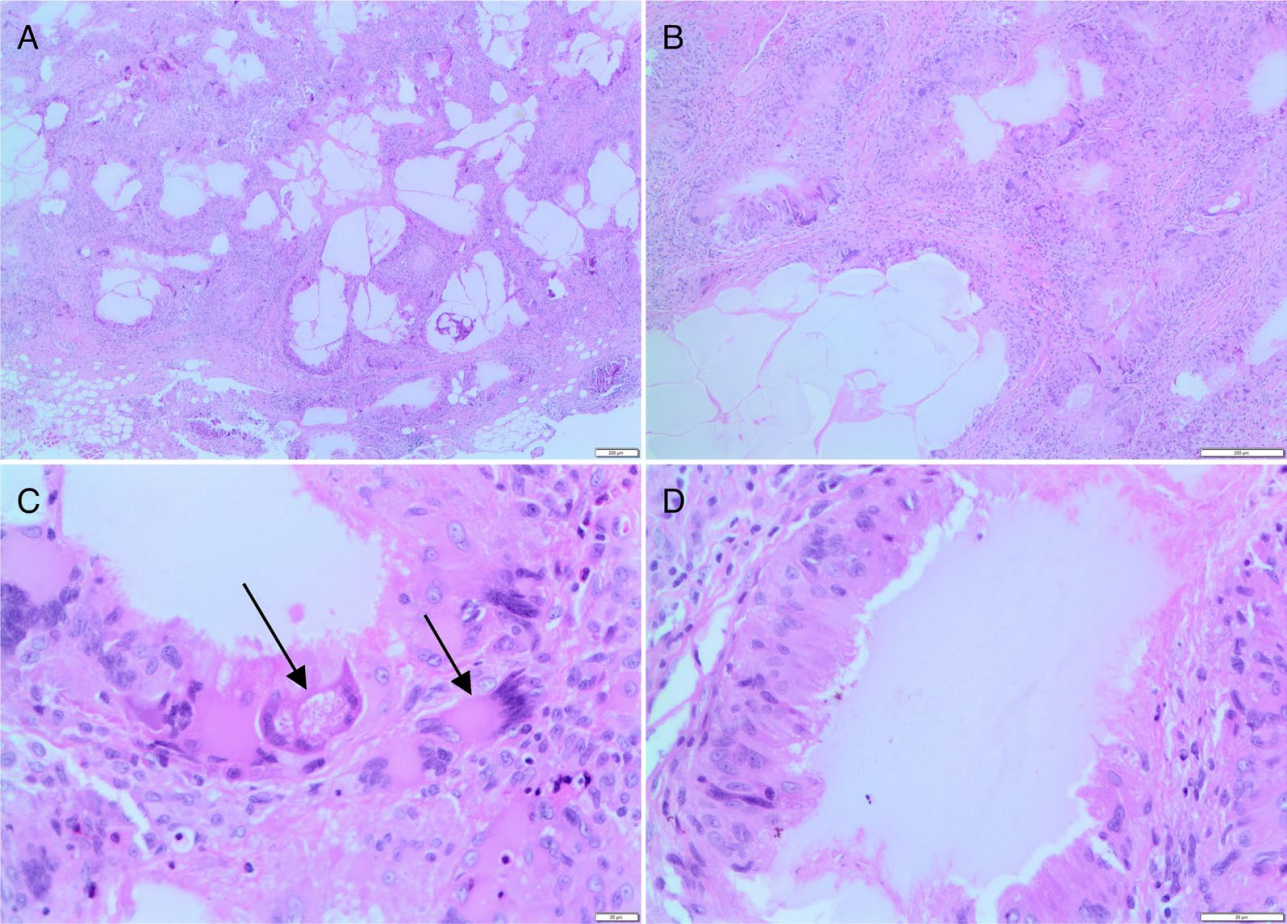
Submucosal fibroadipose tissue almost entirely effaced by multiple empty spaces and large pools of pale staining eosinophilic material (**A**) eliciting a prominent foreign-body giant cell granulomatous response (**B**). Non-necrotizing granulomatous inflammation with numerous multinucleated foreign-body giant cells (**C**, arrowed) surround the pale pink stained material (**D**) (H&E stain; original magnification × 200 (**A**, **B**). × 400 (**C**, **D**)

An incision biopsy of the left side of the upper lip done under local anesthesia comprised three portions of soft tissue measuring 25 × 10 x 5 mm. Histologic examination showed fibrous connective tissue with bundles of skeletal muscle fibers and lobules of adipocytes, devoid of surface epithelium/epidermis and almost entirely effaced by large pools of pale staining eosinophilic material that elicited a prominent foreign-body giant cell granulomatous response (Fig. 1A, B). Numerous multinucleated foreign-body giant cells (Fig. 1C, arrowed) with associated lymphocytes, plasma cells and macrophages, and marked eosinophilia surrounded the pale pink stained material (Fig. 1D). The chronic non-necrotizing granulomatous inflammation infiltrated between bundles of skeletal muscle fibers and in a perineural pattern. Caseation was not evident. Special stains (periodic acid Schiff/Ziehl–Neelsen) showed no specific infective etiological agent. A salivary gland neoplasm was not identified.

### Diagnosis and treatment

The diagnosis was that of a foreign-body granulomatous response to dermal fillers. The patient had Juvéderm VOLIFT™ (17.5 mg/ml hyaluronic acid gel, 0.3 mg/ml lidocaine) Allergan Pharmaceutical (Pty) Ltd. placed 10 months prior, to the midface and peri-oral areas. The midface filler remained soft and impalpable with no adverse reaction to this area. The reaction was treated with a combination of hyaluronidase (Hyalase®, Mylan Epd, LLC), betamethasone (Celestone Soluspan®, Organon LLC) and 5-flurouracil (Fluracedyl®, Teva, LLC) by intra-lesional injection using a fanning technique with a 30G needle (Boulle and Heydenrych 2015; Artzi et al. 2020). The area was treated monthly for 11 consecutive months to achieve total clinical resolution.

The local Human Research Ethics Committee (Medical), WITS, Johannesburg, South Africa, granted ethics clearance (M220282) in accordance with the Declaration of Helsinki (2013).

## Discussion

The placement of any filler product is associated with the risk of an adverse reaction (Dyke et al. 2010; Funt and Pavicic 2013). Complications may either be immediate or delayed, of short or long duration, and mild (seen in most cases) or serious, affecting aesthetics and function (Dyke et al. 2010; Funt and Pavicic 2013). As the need for and the number and range of procedures performed increase, so does the risk, number and scale of adverse reactions (Funt and Pavicic 2013). Generally, adverse reactions are seen in up to 12% of patients, and severe complications are noted in about 1:1600 cases (John and Price 2009).

Dermal fillers are categorized as natural fillers (hyaluronic acid [HA], calcium hydroxyapatite, human-derived collagen, bovine collagen and poly-L-lactic acid) or synthetic fillers (silicone, polyacrylamide, polymethyl methacrylate [PMMA], polyalkylimide, polyvinylhydroxide) (Owosho et al. 2014). They are alternatively classified as biodegradable or non-biodegradable fillers and according to particulate and non-particulate fillers. Biodegradable fillers are further categorized as being of moderate or long duration (Funt and Pavicic 2013). Ideally, fillers should be safe (non-allergenic, non-carcinogenic), stable, easy to use and store, cost-effective, compatible with human tissue, and show little or no adverse reactions, migration and minimal recovery time and inflammatory response. There should also be bulk supply available, long-lasting with slow degradation within the body (Owosho et al. 2014), and easy to remove if necessary (Fernandez-Cossio and Castano-Oreja 2006). There are approximately 160 dermal filler products available worldwide, manufactured by 50 different companies (Funt and Pavicic 2013).

The immediate or early side effects include injection site reactions (pain, edema, erythema, itching, bruising), infection, hypersensitivity, lumps/nodules, asymmetry, defects in contour, skin discoloration, vascular compromise and tissue necrosis (Funt and Pavicic 2013). In addition to edema, nodules, pain and infection, delayed adverse reactions include foreign-body granuloma, migration, infection, immune reactions, tenacious scarring and discoloration and skin compromise (Funt and Pavicic 2013). Evidence-based animal studies using natural dermal filler such as HA and collagen show these to undergo resorption by macrophages and/or giant cells followed by elimination of the filler particles, resulting in clinically visible shrinkage with time (Eversole et al. 2013).

The pathogenetic mechanisms underlying the formation of nodular granuloma formation remain incompletely understood. Fillers acting as adjuvants may result in activation of the innate and adaptive immune responses. Subsequent triggers causing cytokine dysregulation result in macrophage reactivation and granuloma formation. Local trauma, local or distant infection, vaccinations, genetic and molecular variants in host response or immunogenic stimulation from protein contaminants are all implicated (Alijotas-Reig et al. 2013b). Furthermore, particle surface chemistry and surface roughness may trigger heterogeneous host responses to the filler particles (Eversole et al. 2013). It has also been suggested that low molecular weight HA degradation products have higher proinflammatory activity (Farwick et al. 2011). Even though the actual reason for adverse reactions to HA is still unclear, it shows a stronger association with the cross-linking process rather than the source or nucleic acid contaminants (John and Price 2009). The more cross-linked and concentrated the products are, the longer acting it is, thereby increasing the reactivity within the body, leading to a greater risk of inflammation and granuloma formation (Funt and Pavicic 2013).

HA is an inert filler that may persist at the injection site, mimicking a tumorlike nodule (Farahani et al. 2012). Some dermal fillers may cause inflammatory reactions with resultant tissue destruction, which may also mimic a malignant neoplasm (Pinheiro et al. 2019). Patients that fail to disclose having filler procedures coupled with migration of the filler is often a major contributing factor to misdiagnosis. Migration of fillers is not uncommon and is due to high volume and high-pressure injection, muscle movement or gravity. Natural tension lines can also be a contributing factor (Pinheiro et al. 2019; Shahrabi-Farahani et al. 2014).

Reported clinical mimics include adenoma, pleomorphic adenoma, sclerotic minor salivary gland, fibroma, basal cell carcinoma, mucocele, salivary gland tumor and benign soft tissue neoplasm (Table 1) (Eversole et al. 2013; Farahani et al. 2012; Pinheiro et al. 2019; Shahrabi-Farahani et al. 2014; Tamiolakis et al. 2018; Mandel et al. 2010; Davis et al. 2019). Clinical and cytologic/histologic mimics include foreign-body granulomatous reaction (*n* = 24), while purely cytologic/histologic mimics include liposarcoma (*n* = 12) and mucoepidermoid carcinoma (*n* = 2) (Eversole et al. 2013; Pinheiro et al. 2019; Shahrabi-Farahani et al. 2014; Jham et al. 2009; Singh et al. 2010; Davis et al. 2019). The current case is interesting in the clinical concern and subsequent FNA diagnosis suggestive of either a pleomorphic adenoma or a mucoepidermoid carcinoma.

**Table 1:** Reported adverse granulomatous reactions to dermal fillers mimicking salivary gland neoplasia

Reference	Cumulative unique reported cases (*n*)	Filler type	Differential diagnosis	Histologic/cytologic/clinical mimic
Pinheiro et al. (2019)	1	PMMA	Pleomorphic adenoma	Clinical
Eversole et al. (2013)	2	HA	Mucoepidermoid carcinoma	Histologic
Davis et al. (2019)	3	HA	Mucoepidermoid carcinoma	Cytologic
Farahani et al. (2012)	4	HA	Adenoma	Clinical
Shahrabi-Farahani et al. (2014)	5	HA	Sclerotic minor salivary gland	Clinical
Tamiolakis et al. (2018)	6	HA	Salivary gland tumor	Clinical
	7	Liquid silicone	Mucocele	Clinical
Mandel et al. (2010)	8	Silicone	Parotid gland swelling	Clinical

Dermal fillers cause foreign-body reactions, which usually present as asymptomatic swellings that may be associated with pain, pruritus, burning sensation or erythema (Owosho et al. 2014). Table 1 is a summary of previous reports. Most reported patients are female (7 of 8 patients) between 32 and 80 years. All but one of the lesions are asymptomatic and painless. The single painful lesion was present for 2 years becoming symptomatic 3 months prior to the patient seeking medical care. None of the patients had any significant medical history. One patient had a prior smoking history. The lesions all show normal overlying mucosa. A single extra-oral case presented as diffuse cheek swelling with mild cutaneous erythema. Adverse dermal reactions include discomfort, and either discrete or poorly circumscribed lumps/nodules that are either firm or soft and fluctuant, tender or non-tender, mobile and range in size from 0.5 to 2 cm. The manifestation of the adverse reaction following filler placement ranges generally from 2 to 24 months, with one case recurring after 9 years. The adverse reaction in this patient occurred 29 months after filler placement, spontaneously regressed without treatment after 2–3 weeks, and then recurred 9 years later, presenting for 4 weeks without regression. The reason for the delayed response was not apparent, and while the exact mechanism of action for the delayed reaction to the HA fillers is unknown, it most probably is due to structural modification in the cross-linking of the HA filler, increasing the product’s resistance to enzymatic breakdown, resulting in increased longevity of the injected filler (Munavalli et al. 2022).

Adverse reactions to dermal fillers may only develop months following the procedure; thus, patients may have difficulty correlating the reaction to the cosmetic procedure, and hence non-disclosure to the clinician (Owosho et al. 2014). Dermal fillers, however, display distinct recognizable histomorphology, with the host response to the foreign material, often being unique for each material, especially in the sensitive patient (Eversole et al. 2013). The histopathologic pattern is often that of dermal filler lakes, with associated resident macrophages, emigrating monocytes with epithelioid features and multinucleated giant cells (Eversole et al. 2013). Frequently fibroblast activation and significant collagen deposition occur on the periphery of the granulomas.

Adverse reactions are noted to PMMA, HA and silicone, each demonstrating specific histologic features. PMMA shows fragments of loose connective tissue with a capsular organization around numerous spherules of transparent synthetic material of uniform diameter, surrounding foreign-body-type giant cells, areas of fibrosis and a focal intense lymphocytic inflammatory infiltrate, without birefringence properties with fluorescent and polarized microscopy (Pinheiro et al. 2019). The adverse reaction to HA typically shows pools of amorphous hematoxyphilic (basophilic) material enclosed by collagenized connective tissue generally without inflammation and with or without a foreign-body reaction (Farahani et al. 2012), with the Alcian blue and colloidal-iron-positive acid mucopolysaccharides. There may be multiple cystic areas of lakes of HA lined by epithelioid cells (CD68+) forming a foreign-body granuloma. The papillary cystic pattern may be misinterpreted as a low-grade mucoepidermoid carcinoma (Eversole et al. 2013). This is further compounded by acellular material of variable morphology, extracellular matrix or mucin and vacuolated histiocytes (mucocytes) observed on FNA. Adjacent minor salivary gland lobules showing atrophy and sialadenitis in oral and perioral lesions further raise the possibility of a salivary gland neoplasm (Farahani et al. 2012). The presence of silicone filler is marked by numerous empty spaces and vacuolated macrophages (Fig. 1A, B). These fibrous cyst-like formations together with clear acellular droplets may be surrounded by scattered multinucleated giant cells and small foci of inflammation (Fig. 1C, D) (Mandel et al. 2010). Liquid silicone fillers usually show variably sized clear cystic spaces that may contain amorphous eosinophilic material, interspersed within a fibrous connective tissue stroma (Tamiolakis et al. 2018). The spaces may be surrounded by vacuolated epithelioid histiocytes with a signet ringlike appearance intermixed with a few multinucleated giant cells (Tamiolakis et al. 2018), which also is not birefringent with polarized light microscopy.

In a review of 104 published cases of oral foreign-body granulomas due to soft tissue fillers, Tamiolakis et al. (Tamiolakis et al. 2018) found the clinical diagnostic differentia to include mucoceles, benign salivary gland and soft tissue neoplasms for single lip nodules/masses and orofacial granulomatosis, angioedema, and Crohn’s disease for diffuse swellings. In a review of 49 previously reported cases of biomaterial-induced granulomas in the lip and oral cavity, the most common clinical suspicion for lip nodules, and foreign-body reactions seen in patients with a history of dermal filler procedures are salivary gland-related lesions (Jham et al. 2009).

Foreign-body granulomas are the most common histological pattern of the delayed onset dermal filler reactions, with an incidence of between 0.02 and 2.8% (Tamiolakis et al. 2018). Many clinical circumstances such as local infection and host immune responses give rise to granuloma formation with a specific histological appearance for each type of filler (Shahrabi-Farahani et al. 2014; Faria et al. 2014). Despite these distinct histologic appearances for each type of filler material, the identification of the offending agent remains a challenge even for the experienced pathologist (Faria et al. 2014).

Histopathologic identification of the dermal filler is essential for diagnostic, therapeutic and medicolegal reasons. Various techniques aid in the identification of dermal filler materials in tissues. These include energy-dispersive X-ray analysis or microanalysis (EDXA), high-frequency ultrasonography and spectroscopy (Owosho et al. 2014). EDXA identifies the element material composition and aids in the identification of calcium hydroxyapatite dermal filler in tissue samples (Owosho et al. 2014). Silicone is identified by EDXA in a fresh tissue sample and not in paraffin-embedded tissue as the silicone filler material is lost during histologic processing. EDXA cannot be used to identify dermal fillers such as HA, collagen, poly-L-lactic acid and polyacrylamide as these fillers are organic compounds composed of carbon, hydrogen and oxygen. The high-frequency ultrasonography technique is a noninvasive technique that aids in identifying the location and quantity of the filler material. This technique also allows differentiation of natural (temporary) and synthetic (permanent) fillers, which demonstrate hypoechoic and hyperechoic patterns, respectively. This technique unfortunately cannot identify material composition. Spectroscopy is a simple technique based on the principle that molecules absorb or emit specific frequencies characteristic of their chemical bonds (Owosho et al. 2014).

Accurate diagnosis of a foreign-body granuloma response to dermal fillers is challenging to both the clinician and the pathologist due to the prolonged time lapse following the procedure and appearance of the lesion, coupled with patients’ deliberate denial of dermal filler placements (Tamiolakis et al. 2018; Jham et al. 2009). The reported time for the clinical manifestation of a granulomatous reaction following dermal filler placement varies from 9 days to 12 years (Jham et al. 2009). There are many similarities between granulomatous reactions to dermal fillers and salivary gland neoplasms, and thus a combination of the clinical history, examination and tissue biopsy is important in establishing a correct diagnosis. Medical imaging is indicated for carcinoma staging but is of limited use in granulomatous reactions.

The role of FNA cytology (FNAC) in the diagnosis of foreign-body granulomas to dermal fillers is controversial. While some reports show FNAC to be a less invasive, useful method to confirm clinical suspicions of adverse reactions to dermal fillers (Faria et al. 2014), others express many potential diagnostic pitfalls in the cytologic evaluation of reactions to dermal fillers (Davis et al. 2019). The latter sentiment is reinforced in our case, which was misdiagnosed as a pleomorphic adenoma or mucoepidermoid carcinoma on FNAC.

Interestingly, patients with a history of dermal filler use have demonstrated side effects with the mRNA COVID-19 vaccine. The Centers for Disease Control and Preventions (CDC) have thus highlighted clinical considerations for mRNA COVID-19 vaccine use and its likely consequence in patients with a history of dermal filler use (https://www.cdc.gov/vaccines/covid-19/info-by-product/clinical-considerations.html). This may contribute to an increase in adverse reactions to dermal fillers. Even though this is very infrequent, temporary and treatable with corticosteroid therapy, knowledge and recognition of this possible reaction by both the clinician and patient is important (https://www.cdc.gov/vaccines/covid-19/info-by-product/clinical-considerations.html).

In the phase 3 trial of 30 000 mRNA-vaccinated subjects (Moderna COVID-19 vaccine), three patients with dermal fillers (1 lip, 2 cheek) showed moderate facial swelling as an adverse reaction (https://www.healio.com/news/dermatology/20210119/qa-reaction-to-facial-fillers-may-be-seen-with-moderna-covid19-vaccine). No specific filler type was associated with this side effect. The patient with the lip filler reported a similar reaction previously following a flu vaccine. This reaction may be because most dermal fillers are composed of hyaluronic acid, a natural substance and a component of our collagen, which retains fluid. In an attempt to create natural immunity to a virus following vaccination, the immune system reacts by sending healing cells to the area, resulting in swelling. In a patient with dermal fillers undergoing an immune response, the increased hyaluronic acid in the dermal filler could result in swelling due to the ability of hyaluronic acid to retain water (https://www.healio.com/news/dermatology/20210119/qa-reaction-to-facial-fillers-may-be-seen-with-moderna-covid19-vaccine).

Knowledge of facial anatomy, different injection techniques, the various properties of the different filler products and their indications, history of filler placement and the patients’ needs and expectations are essential in reducing the risk of adverse outcomes (Koli and Davda 2016). Pre-procedural assessment should be cognizant not only of a complete medical history but also of the process of aging of the face, especially the different times and rate in the various facial areas. The medical history should be attentive to bleeding disorders, uncontrolled hypertension, drug usage such as anticoagulants and blood thinners. A thorough understanding of the product used such as the gel hardness, cross-linkage, particles per milligram, monophasic or biphasic nature, shelf life and nature of additives such as lignocaine is crucial as this allows for ideal filler placement (Koli and Davda 2016). The correct depth of filler placement is influenced by the hardness of the product, for example, the harder the filler material, the deeper it should be injected (Funt and Pavicic 2013). This underwrites an understanding of the treatment of the adverse outcomes following dermal filler procedures.

The global demand for dermal filler placement necessitates a focus on patient safety. The recently updated 10-point plan for procedural safety in soft tissue filler treatments provides treating physicians with methodical strategy for avoiding and managing adverse reaction (Heydenrych et al. 2021). Patients should be well versed of their responsibility to disclose information of filler placement should a soft tissue reaction develop.

## Conclusions

The clinical and histological diagnosis of an adverse granulomatous reaction to a dermal filler is often challenging for diverse reasons, including COVID-19 vaccinations. An awareness of this manifestation mimicking a salivary gland neoplasm is significant to ensure the correct diagnosis and treatment. Patient education regarding the name and nature of the injected filler material and the possibility of adverse reactions is important.

## Data Availability

Not applicable.

## Abbreviations

ASPS: American Society of Plastic Surgeons
FNA: Fine needle aspiration
Fig.: Figure
mg/ml: Milligrams/milliliters
(Pty) Ltd: Proprietary limited
Epd: Environmental protection division
LLC: Limited liability company
30G: 30 Gauge
WITS: University of the Witwatersrand
HA: Hyaluronic acid
PMMA: Polyacrylamide, Polymethyl methacrylate
*n*: Number of samples
EXDA: Energy-dispersive x-ray analysis or microanalysis
FNAC: Fine needle aspiration cytology
mRNA: Messenger ribonucleic acid
COVID-19: Corona virus disease-19
CDC: Centers for Disease Control and Preventions
